# Supplementary material for: Replacing murine insulin 1 with human insulin protects NOD mice from diabetes
Source: PLoS One. 2019 Dec 10;14(12):e0225021. doi: 10.1371/journal.pone.0225021 (PMC6903741; doi:10.1371/journal.pone.0225021)
Supplement: S1 Fig — (PDF) [file pone.0225021.s001.pdf]

S1 Figure
Sequence of the homology-directed repair (HDR) template

```
chr19:52263923-52265456 (mm10)
ccaatgagtgggctacggggtttgtgaaaggagagatggagaaggagggaccattaa
gtaccttgctgcctgagttctgctttcctcctccctctgagggtgagctgggatctc
atctgagttaagggcccagctatcaatgggaactgtgaaacagtccaagggacatca
atattaggtccctaacaactgcagtttcctggggaatgatgtggaaaatgctcagcc
aaagatgaagaaggtctcaccttctgggacaatgtcccctgctgggaactggttcat
caggccatctggtcccttattaagactataataaccctaagactaagtagatgtgtt
gatgtccaatgagtgctttctgcagacctagcaccaggcaagtgtttggaaactgca
gcttcagcccctctggccatctgcctacccaccccacctggagaccttaatgggcca
aacagcaaagtccagggggcagagaggaggtactttggactataaagctggtgggca
tccagtaaccccagcccttagtgaccagctataatcagagaccatcagcaagcagg
tatgtactctcctctttgggcctggctcccCAGCCAAGACTCCAGCGACTTTAGGGA
GAATGTGGGCTCCTCTCTTACATGGATCTTTTGCTAGCCTCAACCCTGCCTATCTTT
CAGGTCATTGTTTCAACATGGCCCTGTGGATGCGCCTCCTGCCCCTGCTGGCGCTGC
TGGCCCTCTGGGGACCTGACCCAGCCGCAGCCTTTGTGAACCAACACCTGTGCGGCT
CACACCTGGTGGAAGCTCTCTACCTAGTGTGCGGGGAACGAGGCTTCTTCTACACAC
CCAAGACCCGCCGGGAGGCAGAGGACCTGCAGGTGGGGCAGGTGGAGCTGGGCGGGG
GCCCTGGTGCAGGCAGCCTGCAGCCCTTGGCCCTGGAGGGGTCCCTGCAGAAGCGTG
GCATTGTGGAACAGTGCTGCACCAGCATCTGCTCCCTCTACCAGCTGGAGAACTACT
GCAACTAAGGCCCACCTCGACCCGCCCCACCCCTCTGCAATGAATAAAACTTTTGAA
TAAGCACCAAAAAaaagagttctataatgaatgaaaaaggattgtgtatatagacat
ctttttctctggcatttattgtcatgttagcatactattaaaccattgttaggttgg
atgattatataatcatgtatgaagcttgtgataaaacaccaggaataattcaagtat
ctggaattctgcttcctgcccaagaaggtaggcaaccgtgtaaatgccactgaagct
actagtctaaaagtgagttatctctgtctttgtcttacccccctgatgctgtgataaa
accctgacaagagcaactgactcctgagaggaaggtttattctagctcacaattcca
ggttacaaacagtccatccgtagcaggggagtcacagcaacaggaacctcagggaac
tgctcctattatccccacaatcaagaatagtgaccaataaataagtggatcttttct
c
```

Start codon
Humanised region
Region of homology between human and mouse
Stop codon

INS transcript = 333bp
701bp 5' homology with murine chr19
559bp 3' homology with murine chr19 (including 53bp that overlaps with the end of the human INS coding region)
