# Supplementary material for: Replacing murine insulin 1 with human insulin protects NOD mice from diabetes
Source: PLoS One. 2019 Dec 10;14(12):e0225021. doi: 10.1371/journal.pone.0225021 (PMC6903741; doi:10.1371/journal.pone.0225021)

S2 Fig. Human insulin is localised to the pancreatic islets in NOD.HuPI mice

Pancreas sections from NOD.HuPI wildtype (A,B) and KI/KI (C,D) mice were stained with anti-human proinsulin/C-peptide (GN-ID4). Positive staining is indicated in red. DAPI staining in blue.

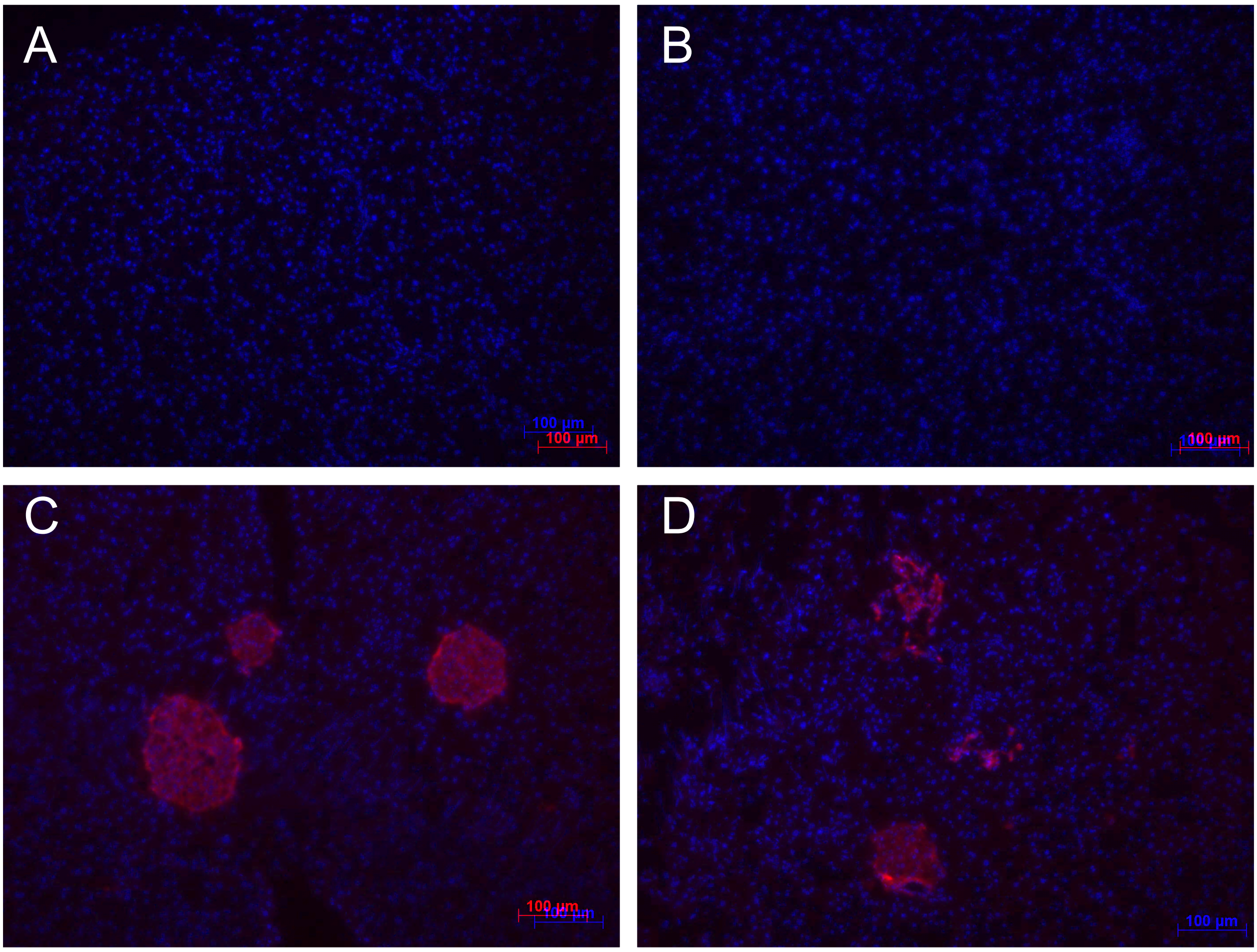

Supplement: S2 Fig — (PDF) [file pone.0225021.s002.pdf]
