# Supplementary material for: Replacing murine insulin 1 with human insulin protects NOD mice from diabetes
Source: PLoS One. 2019 Dec 10;14(12):e0225021. doi: 10.1371/journal.pone.0225021 (PMC6903741; doi:10.1371/journal.pone.0225021)

S3 Fig. NOD.HuPI mice exhibit delayed insulitis (100d).
Representative images of pancreas sections from 100d-old NOD.HuPI wildtype (A-F), and KI/KI (G-L) mice, stained with haematoxylin and eosin. Photos taken at 100x magnification.

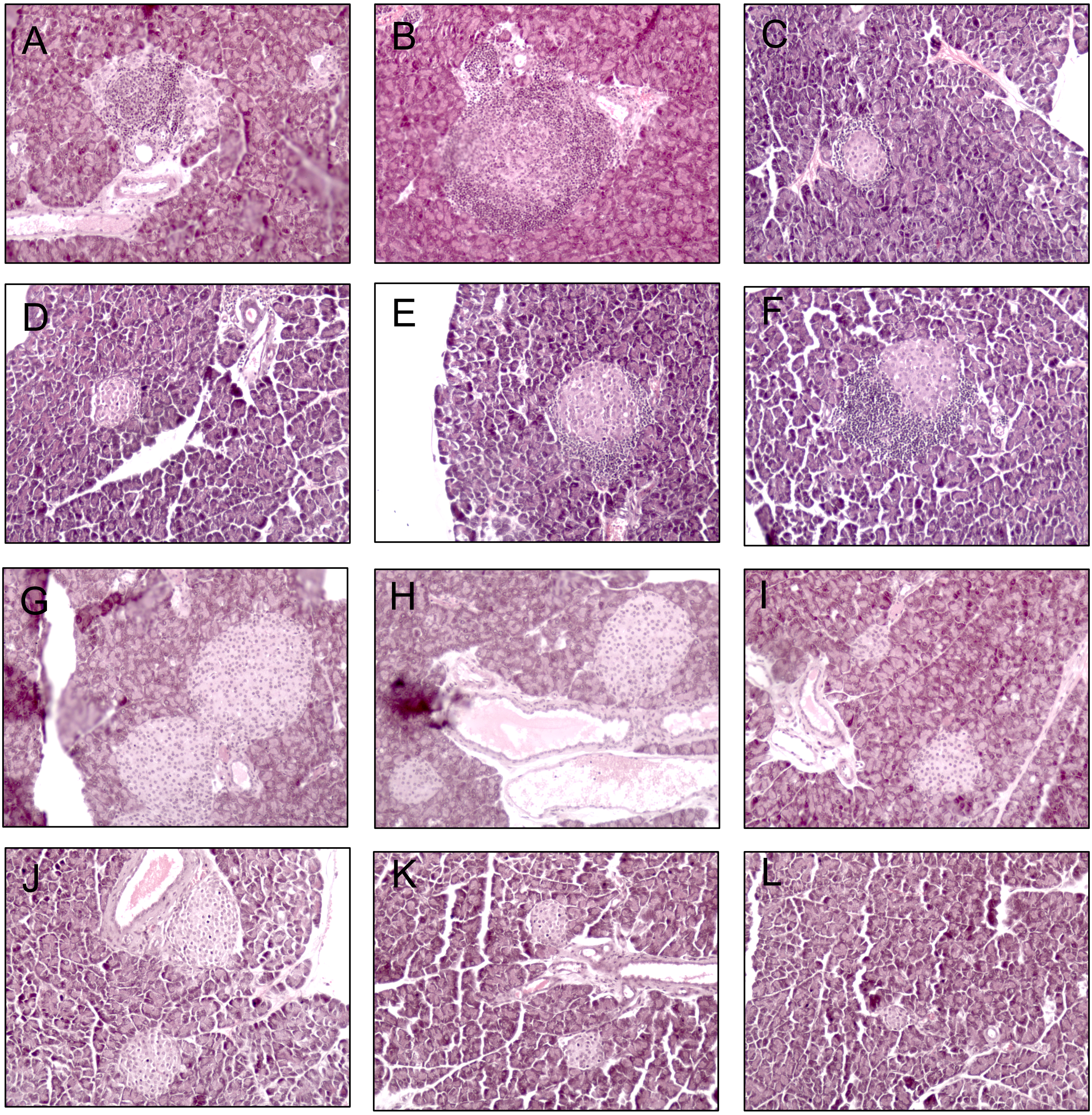

Supplement: S3 Fig — (PDF) [file pone.0225021.s003.pdf]
