# Supplementary material for: Replacing murine insulin 1 with human insulin protects NOD mice from diabetes
Source: PLoS One. 2019 Dec 10;14(12):e0225021. doi: 10.1371/journal.pone.0225021 (PMC6903741; doi:10.1371/journal.pone.0225021)

S4 Fig. NOD.HuPI mice exhibit delayed insulitis (300d).
Representative images of pancreas sections from 300d-old NOD.HuPI wildtype (A-B), and KI/KI (C-L) mice, stained with haematoxylin and eosin. Photos taken at 100x magnification.

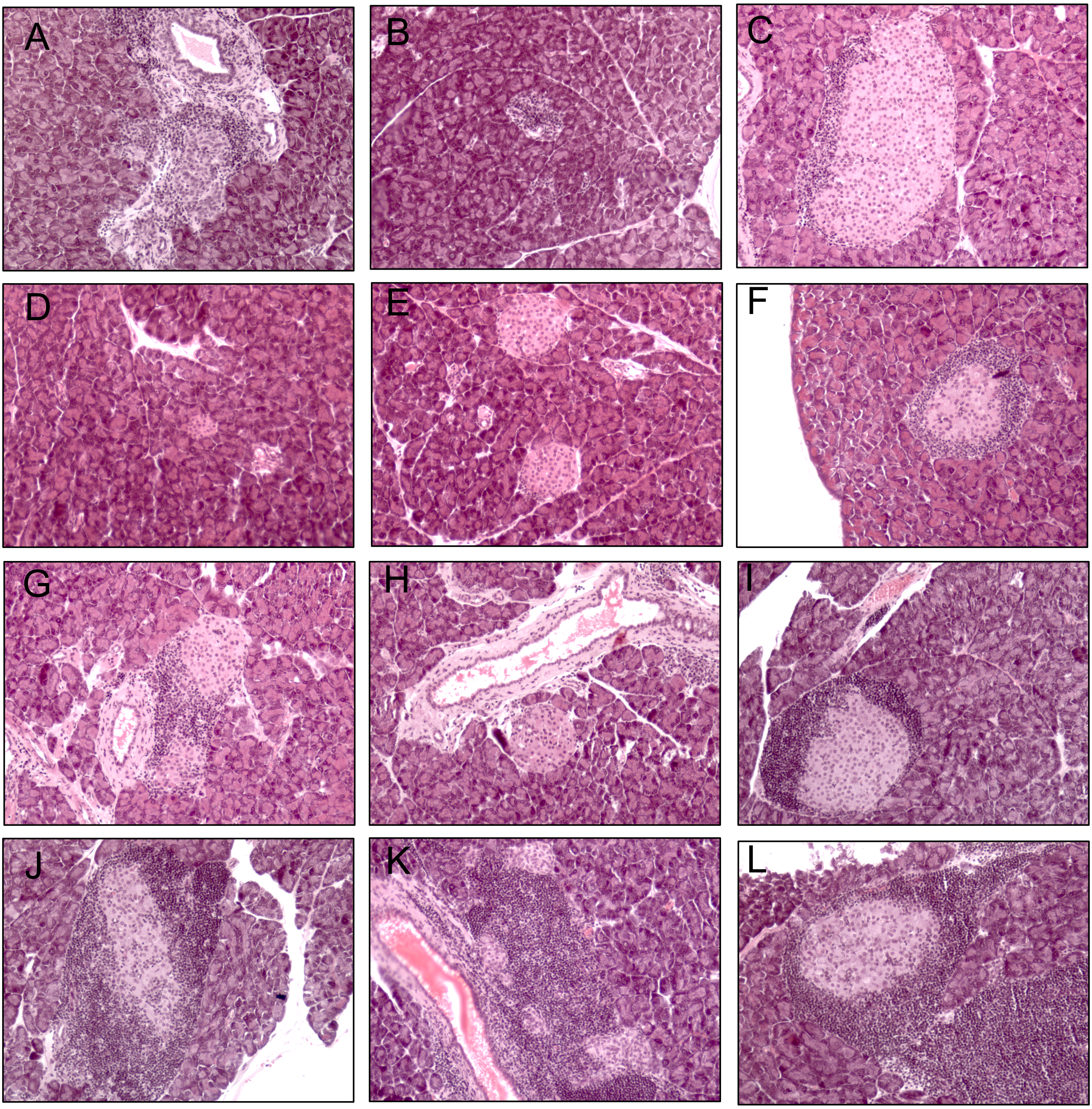

Supplement: S4 Fig — (PDF) [file pone.0225021.s004.pdf]
