# Supplementary material for: Replacing murine insulin 1 with human insulin protects NOD mice from diabetes
Source: PLoS One. 2019 Dec 10;14(12):e0225021. doi: 10.1371/journal.pone.0225021 (PMC6903741; doi:10.1371/journal.pone.0225021)
Supplement: S1 Table — (PDF) [file pone.0225021.s005.pdf]

S1 Table. Production of human insulin knock-in mice by CRISPR/Cas9 mutagenesis.

sgRNAs were designed to target the 5’ and 3’ regions of *Ins1* (listed below; PAM site underlined). Two combinations of sgRNAs were used as shown in the table. NOD/Lt embryos were microinjected cytoplasmically with 30 ng/ul Cas9 mRNA, 15 ng/ul each sgRNA and 30 ng/ul linearized homology directed repair template (See S1 Fig). They were incubated with or without 50 μM SCR7 (a NHEJ inhibitor) overnight prior to transfer into recipients. Offspring were screened by PCR for the presence of the human *INS* gene before further characterisation.

Table 1A Summary of guides and offspring

Injection #	5’ sgRNA	3’ sgRNA	Scr7 incubation	# *INS* positive / # pups
1	1	4	+	0/14
2	1	4	-	1/12
3	1	3	+	4/25

Table 1B Summary of sgRNA sequences

sgRNA name	Sequence	On target score*	Off target score*
sgRNA#1	TTGTTTCAACATGGCCCTGT<u>TGG</u>	62	47
sgRNA#3	CGCCAAGGTCTGAAGGTCCC<u>CGG</u>	50	67
sgRNA#4	CCGGGCCACCTCCAACGCCA<u>AGG</u>	47	71

*On target and off target score calculated at idtdna.com. Score out of 100 where a higher score has a higher likelihood of being on target and less likelihood of off target cutting.
