# Supplementary material for: Replacing murine insulin 1 with human insulin protects NOD mice from diabetes
Source: PLoS One. 2019 Dec 10;14(12):e0225021. doi: 10.1371/journal.pone.0225021 (PMC6903741; doi:10.1371/journal.pone.0225021)
Supplement: S2 Table — (PDF) [file pone.0225021.s006.pdf]

S2 Table. Molecular confirmation of *INS* knockin.

Five founder mice were identified with correct sized products across the *INS* gene by PCR (Human insulin). The insert sequences in these mice were further characterised to determine whether they had inserted in the correct genomic location by identifying correct sized PCR products at the 5’ (Site of integration 5’) and 3’ (Site of integration 3’) end of the insert construct with primers flanking the homology arms of the HDR construct. PCR products were sequenced across the INS gene to confirm the presence of the correct sequence. + = presence of correct sized PCR product. - = absence of correct sized PCR product. Coloured shading indicates positive result. Grey shading indicates negative result. PCR products are indicated on the schematic. Blue line/boxes indicate sequence within the HDR construct. Flanking genomic sequence is indicated in green and orange.

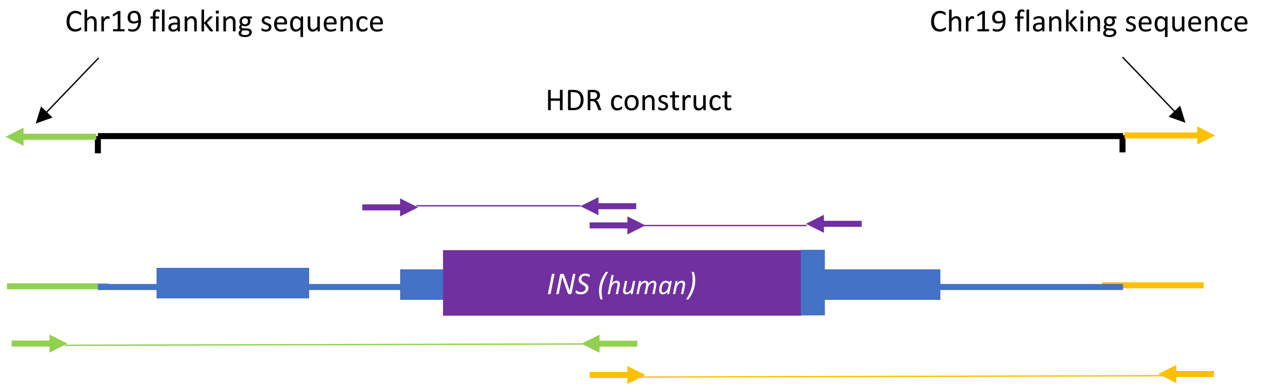

Founder #	Human insulin	Site of integration 5’	Site of integration 3’	Sequencing results
25	+	+	+	Recombination within INS resulting in retention of mouse sequence
41	+	+	-	ND
44	+	+	+	Missense mutation in INS
48	+	-	-	ND
52	+	+	+	Correct
