# Supplementary material for: Replacing murine insulin 1 with human insulin protects NOD mice from diabetes
Source: PLoS One. 2019 Dec 10;14(12):e0225021. doi: 10.1371/journal.pone.0225021 (PMC6903741; doi:10.1371/journal.pone.0225021)
Supplement: S3 Table — (PDF) [file pone.0225021.s007.pdf]

S3 Table. Genotype ratios in offspring of NOD.HuPI mice.

Sex and genotype ratios were as expected in litters from the founder mouse backcrossed to NOD/Lt. Totals from three litters.

Founder #52 (female) x NOD/Lt (male)

Ratio	Observed	Expected	X^2	df	P
Female:Male	11:11	11:11	0	1	> 0.5
KI/+:+/+	12:10	11:11	0.18	1	> 0.5

Sex and genotype ratios were as expected from the first generation of intercross litters. Totals from two litters.

KI/+ (female) x KI/+ (male)

Ratio	Observed	Expected	X^2	df	P
Female:Male	8:16	12:12	2.67	1	> 0.1
KI/KI:KI/+:+/+	11:9:4	6:12:6	5.58	2	> 0.05
