# Supplementary material for: Replacing murine insulin 1 with human insulin protects NOD mice from diabetes
Source: PLoS One. 2019 Dec 10;14(12):e0225021. doi: 10.1371/journal.pone.0225021 (PMC6903741; doi:10.1371/journal.pone.0225021)
Supplement: S4 Table — (PDF) [file pone.0225021.s008.pdf]

S4 Table Insulin-specific primers

Gene	F primer	R primer	Anneal temp	cDNA Size	gDNA Size
INS	CGCAGCCTTTGTGAACCAAC	CCACCTGCCCCACCTGCAGG	55	134bp	134bp
Ins1	TTCTACACACCCAAGTCCCG	AAGTTTTATTCATTGCAGAGGGGTG	68	227bp	227bp
Ins2	CTTCCTCTGGGAGTCCCAC	TGCAGTAGTTCTCCAGCTGG	55	287bp	775bp
Actb	CGGTTCCGATGCCCTGAG	TGATCCACATCTGCTGGAAGG	55	312bp	527bp

Cloneamp HiFI Premix was used to amplify cDNA with 0.2μM primers. PCR conditions: 35 cycles of 98°C 10 sec, annealing temp 15sec, 72°C 10sec, followed by 72°C 7min.
