# Supplementary figures and images for: Replacing murine insulin 1 with human insulin protects NOD mice from diabetes
Source: PLoS One. 2019 Dec 10;14(12):e0225021. doi: 10.1371/journal.pone.0225021 (PMC6903741; doi:10.1371/journal.pone.0225021)

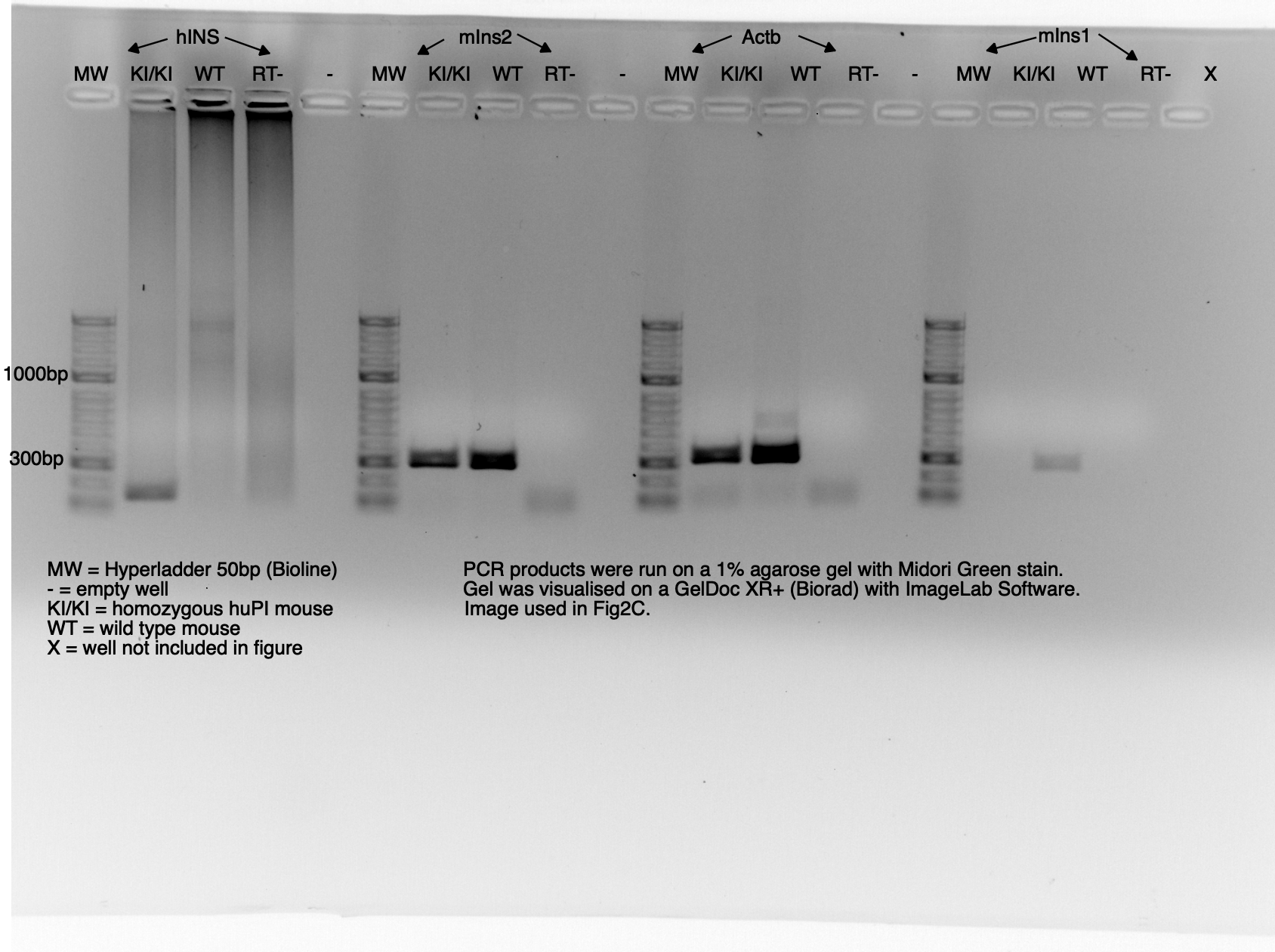

Supplement: S1 Raw Image — (TIF) [file pone.0225021.s009.tif]
