# Supplementary material for: Replacing murine insulin 1 with human insulin protects NOD mice from diabetes
Source: PLoS One. 2019 Dec 10;14(12):e0225021. doi: 10.1371/journal.pone.0225021 (PMC6903741; doi:10.1371/journal.pone.0225021)
Supplement: Attachment — Submitted filename: R2R.docx [file pone.0225021.s010.docx]

**“Replacing murine insulin with human insulin protects NOD mice from diabetes”**

**Response to the reviewers’ comments**

Dear Editor,

First, we’d like to thank the Academic Editor and the reviewers for their constructive and

thoughtful comments on this manuscript. Below, we address each of the points raised. The

person/people who made these comments is/are show in in parenthesis. The comment, or a

precis of it, is shown in **bold** and our response in plain text. The changes to the manuscript text

are highlighted in red text on the tracked change version.

**1. (Academic Editor and Reviewer 1) Are there any off-target effects of the CRISPR/Cas9?**

We are confident that the protection that we observe in NOD mice is not attributable to off-target

genomic mutations caused by CRISPR/Cas 9 for the following reasons.

1. The mutation was back crossed to NOD mice for three generations. Each back cross

reduces the load of putative off target mutations by 50%, so after three generations

>87% of putative off-target mutations would have been lost.

2. There was no difference in the diabetes incidence of mice from different branches of

the pedigree, suggesting that the phenotype was due to the alteration of the INS1 locus

not elsewhere in the genome. We saw no difference between litters; if putative

mutations were segregating and responsible for the phenotype, we would have

expected to see differences between litters which we did not. The wildtype mice were

a mix of littermate controls and NOD mice from our colony. There were no differences

between these two groups of control mice. If there was an off-target or background

effect, then we would have seen it there too.

3. Off-target effects of CRISPR have been reported to be lower in vivo than similar

experiments in vitro [1]. Furthermore, the putative CRISPR/Cas9 introduced

mutations should be considered in the context of the background mutation rate per

mammalian generation has been estimated to be 70-175 de novo mutations per

diploid genome per generation [2, 3].

4. The single guide RNAs had relatively high off-target scores on IDT’s algorithm

(https://sg.idtdna.com/site/order/designtool/index/CRISPR_SEQUENCE). The

sgRNAs used that led to the mouse in the study had off target scores of 47 and 67,

indicating that they have a modest risk of off target effects. These scores are now

included in Supplementary Table 1B.

Cover letter for revisions

5. In addition to the points raised above and given insulin’s well-established role in the

immune pathogenesis of diabetes, both in humans and NOD mice it is extremely

unlikely that protection from diabetes would develop in CRISPR-modified mice due to

a mutation in an unrelated gene.

The possible role of off target mutations is now discussed on page 5, lines 97-100 and in the

Discussion (page 13, lines 256-265).

**2.(Reviewer 1) What do the authors mean by 'most of the amino acid coding region' being**

**replaced (l142, p8)?**

Because the final 16 amino acids in the A-chain of murine insulin 1 and human insulin are

identical this part of the gene was not modified. This has now been clarified on page 9, lines 163-

165.

**3. (Reviewer 1) The antibodies used for ELISA and IHC to detect mouse vs human insulin:**

**how certain are the authors that there is no cross-reactivity?**

We have used well established and characterized mAbs and appropriate controls to exclude the

possibility of any cross reactivity between mouse and human (pro)insulin.

1. For IHC, the mAb used for histology has been extensively characterized and shown to be

specific for human insulin [4]. Furthermore, we did not detect any staining in sections

from mice that did not express human insulin.

2. For ELISAs we used a commercial ELISA kit from Mercodia ELISAs which is specific for

human insulin. The mAbs used in the ELISA assays for mouse insulin do cross react with

human and murine insulin. However, human insulin can be measured without cross

reactivity with murine insulin. To determine the concentration of murine insulin the

human insulin component is subtracted, according to the manufacturer’s protocol. This is

now described more clearly in Methods section.

**4.(Reviewer 2) How do the authors explain the fact that KI/+ mice have the same**

**incidence of T1D as KI/KI mice?**

The simplest interpretation of this observation is that the expression of human insulin, in place of

murine insulin 1 induces dominant protection against autoimmune diabetes. At this stage it is not

known whether this is mediated by a Treg population, or impacts upon thymic selection, or

another pathway. A thorough analysis of the mechanism(s) underlying the reduced incidence of

autoimmune diabetes in this model would require several years work and is therefore beyond

the scope of the current manuscript.

**5. Discuss more the similarities and differences in the amino acid sequence between**

**mouse ins1, ins2 and human insulin, with regards B:9-23 and Maki Nakayama’s paper in**

**Nature 2004**

The amino acid sequence of insulin B9-23 epitope is identical between murine insulin 1, murine

insulin 2 and human insulin (see Figure 1). All have the pivotal B16 tyrosine residue that was

mutated to alanine in Dr Nakayama’s 2005 Nature paper (Ref No 9 in the manuscript). Figure 1

highlights that the most difference in amino acid sequence are found in the C-peptide of

proinsulin. This is consistent with reports that this region, in addition to B9-23, harbors epitopes

important for the immune mediated destruction of beta cells in the NOD mouse model [5]. This is

now discussed in the Discussion (p14-15, lines 300-308).

**6. The authors need to measure mouse ins1 and 2 as well human insulin in the thymus of**

**+/+, KI/+ and KI/KI mice.**

As noted above a thorough analysis of the mechanism of diabetes suppression in this model is

beyond the scope of the current manuscript. From our genetic analysis we know that the HuPI KI

mice have human insulin in place of murine insulin 1. For this reason, KI/KI mice will not have

any murine insulin 1 in the thymus. We have not modified the murine insulin 2 locus, so this will

not be altered. We assume that human insulin, like murine insulin 1, will be almost exclusively

detected in the pancreatic beta cells consistent with the data we have presented.

**7. (Reviewer 2) Statistics are missing from the M&M section**

Thank you for drawing this to our attention, we have now included a description of the statistical

analysis into the relevant parts of the Methods section.

**8. (Reviewer 2) The authors should back-up the protein expression data of human insulin**

**with quantitative RNA analyses**

With respect, we disagree. We have already shown that mRNA for human insulin is expressed in

the islets of the HuPI mice (Figure 2C). We have also shown that both human insulin protein and

C-peptide are expressed. In our view, the critical point is that human insulin and C-peptide are

expressed, albeit at variable concentrations. In our view measuring the mRNA levels will not give

further insights above what have already be reported.

**9. (Reviewer 2) Could the authors:**

**Purify splenocytes from diabetic +/+, KI/+ and KI/KI mice and transfer them to NOD.Scid?**

**Evaluate Treg frequency and numbers and perhaps perform Treg transfers from KI/KI**

**mice to +/+?**

We thank Reviewer 2 for these helpful suggestions. We are planning a manuscript which will

address the mechanism(s) by which HuPI mice are protected from diabetes. However, to come to

a clear conclusion regarding the mechanism is beyond the scope of this manuscript. Here our aim

is to report the generation and evaluation of HuPI mice.

**10. Could they discuss other similar models (ref 33) further and perhaps how their model**

**could be improved, for example by expressing human HLA?**

A more detailed discussion of other models has now been included in the Discussion (p15-16 line

321-338). We also discuss the further development of this model, including the expression of

HLA and human (pro)insulin specific TCRs.

**11. It is not clear how the PCR analyses were done (lines 168-170).**

Thank you for drawing this to our attention. In addition to the primers’ sequences we have now

included more details about how the PCR was performed in the Materials and Methods section of

the manuscript (p6, line 113-120).

**References cited**

1. Yoshimi K, Kaneko T, Voigt B, Mashimo T. Allele-specific genome editing and correction of

disease-associated phenotypes in rats using the CRISPR-Cas platform. Nature communications.

2014;5:4240. Epub 2014/06/27. doi: 10.1038/ncomms5240. PubMed PMID: 24967838; PubMed

Central PMCID: PMCPMC4083438.

2. Nachman MW, Crowell SL. Estimate of the mutation rate per nucleotide in humans.

Genetics. 2000;156(1):297-304. Epub 2000/09/09. PubMed PMID: 10978293; PubMed Central

PMCID: PMCPMC1461236.

3. Roach JC, Glusman G, Smit AF, Huff CD, Hubley R, Shannon PT, et al. Analysis of genetic

inheritance in a family quartet by whole-genome sequencing. Science. 2010;328(5978):636-9.

Epub 2010/03/12. doi: 10.1126/science.1186802. PubMed PMID: 20220176; PubMed Central

PMCID: PMCPMC3037280.

4. Asadi A, Bruin JE, Kieffer TJ. Characterization of Antibodies to Products of Proinsulin

Processing Using Immunofluorescence Staining of Pancreas in Multiple Species. J Histochem

Cytochem. 2015;63(8):646-62. Epub 2015/07/29. doi: 10.1369/0022155415576541. PubMed

PMID: 26216140; PubMed Central PMCID: PMCPMC4530395.

5. Levisetti MG, Lewis DM, Suri A, Unanue ER. Weak Proinsulin Peptide–Major

Histocompatibility Complexes Are Targeted in Autoimmune Diabetes in Mice. Diabetes.

2008;57(7):1852-60. doi: 10.2337/db08-0068.
